# On the Reciprocally Causal and Constructive Nature of Developmental Plasticity and Robustness

**DOI:** 10.3389/fgene.2018.00735

**Published:** 2019-01-10

**Authors:** Daniel B. Schwab, Sofia Casasa, Armin P. Moczek

**Affiliations:** Department of Biology, Indiana University Bloomington, Bloomington, IN, United States

**Keywords:** developmental genetics, relaxed selection, cryptic genetic variation, niche construction, symbiosis

## Abstract

Exposure to environmental variation is a characteristic feature of normal development, one that organisms can respond to during their lifetimes by actively adjusting or maintaining their phenotype in order to maximize fitness. Plasticity and robustness have historically been studied by evolutionary biologists through quantitative genetic and reaction norm approaches, while more recent efforts emerging from evolutionary developmental biology have begun to characterize the molecular and developmental genetic underpinnings of both plastic and robust trait formation. In this review, we explore how our growing mechanistic understanding of plasticity and robustness is beginning to force a revision of our perception of both phenomena, away from our conventional view of plasticity and robustness as opposites along a continuum and toward a framework that emphasizes their reciprocal, constructive, and integrative nature. We do so in three sections. Following an introduction, the first section looks inward and reviews the genetic, epigenetic, and developmental mechanisms that enable organisms to sense and respond to environmental conditions, maintaining and adjusting trait formation in the process. In the second section, we change perspective and look outward, exploring the ways in which organisms reciprocally shape their environments in ways that influence trait formation, and do so through the lens of behavioral plasticity, niche construction, and host–microbiota interactions. In the final section, we revisit established plasticity and robustness concepts in light of these findings, and highlight research opportunities to further advance our understanding of the causes, mechanisms, and consequences of these ubiquitous, and interrelated, phenomena.

## Introduction

Developmental plasticity is commonly defined as a single genotype’s ability, or the ability of an individual organism, to adjust aspects of phenotype expression in response to changes in environmental conditions (Pfennig et al., 2010). Developmental robustness, then, is meant to capture the opposite: phenotype expression that is robust or insensitive to changes in the environment (Moczek, 2009). In many ways, this captures an intuitive dichotomy, or perhaps better, extremes along a continuum. Among morphological traits in animals, for instance, secondary sexual traits and ornaments tend to be highly sensitive to variation in environmental conditions such as nutrition, whereas the relative sizes of genitalia or the central nervous system are remarkably robust, with traits such as wings or legs being somewhere in between (Emlen et al., 2012). This characterization is meaningful because, as we will suggest throughout the remainder of this manuscript, the level of biological organization – in this case, external morphology – is made specific and is maintained across comparisons. But we will also attempt to demonstrate that this is about where the simplicity ends. Specifically, in the sections that follow this introduction, we will explore three major complications that arise as we try to compare and contrast the biology of developmental plasticity with that of robustness, and their respective implications for evolution.

First, we will make the case that robustness in development need not be equated with *in*sensitivity to environmental variation. Instead, we will highlight how robust phenotypic outputs are made possible across levels of biological organization through the environment-dependent adjustment of developmental and physiological processes. From blood sugar levels maintained in the face of variation in nutritional inputs and physiological demands, to body size-independent organ growth as seen in the central nervous system or genitalia of many animals, developmental and physiological systems seem to exercise remarkable plasticity to enable a robust phenotype. And we will make the opposite case as well: many of the most extreme cases of environment-sensitive development, whether it occurs in response to nutritional variation, seasons, or conspecific densities, and while giving rise to striking changes in phenotypes across environments, simultaneously exhibit remarkably robust behavior on the level of the underlying developmental switch mechanisms and the subsequent developmental programs they trigger. In the honey bee, larval nutrition either does or does not contain high protein and royal jelly and accordingly, larvae reliably either follow a worker or queen fate (Evans and Wheeler, 1999). Similarly, conspecific densities either do or do not sum up above a critical threshold, and then *Schistocerca* nymphs become fated to develop into a gregarious or solitary morph (Simpson et al., 2011). Put another way, for plasticity on the level of the phenotype (morphology) to be fitness enhancing, it requires robust association between inducing environments, and the developmental responses they trigger.

Second, our ability to distinguish between the biology of plasticity and that of robustness (i.e., the degree of plasticity) is complicated further when we consider the actual identity of the underlying developmental mechanisms. We will posit that there is no such thing as a gene or a pathway or a hormone for plasticity, or for robustness. Instead the same genes, pathways, hormones, but most importantly the conversations between them, appear to enable both phenomena. Instead we propose that it is, for the lack of a better metaphor, the exact nature of these conversations that seem to make the difference between a developmental product that is robust, and one that is plastic.

Lastly, we aim to extend our investigation of the biology of plasticity and robustness into dimensions typically not considered in the development and *evo devo* literature – the notion that important components of the selective environment to which an organism responds to, whether in plastic or robust ways, are themselves at least in part shaped by the organism itself, for example through its habitat choices, its environment-modifying behaviors, or the other organisms (such as microbial symbionts) with which it associates. We will show that all of these processes contain both robust and plastic components themselves, and through their execution enable robustness and plasticity at other levels of biological organization. Collectively, our goal in this review is to disentangle the reciprocally causal and constructive interdependencies of developmental plasticity and robustness, one layer at a time, from genes and pathways, to organs and organisms, to constructed niches and symbioses. Though this interdependency between plasticity and robustness is broadly applicable to unicellular and multicellular organisms, we illustrate these points using insights derived primarily from the study of insects and other animal taxa, and with particular reference to work on horned dung beetles in the genus *Onthophagus*. We will begin by looking inward, and by examining the means by which organisms and their component parts respond to changes in environmental conditions.

## Looking Inward: When Environments Shape Developmental Systems

Attempts to elucidate the endogenous developmental mechanisms underlying plastic and robust phenotypes have long been informed by genetic and physiological approaches, with more recent advances emerging from the advent and widespread use of genomic, transcriptomic, epigenetic, and gene network approaches. However, the results emerging from these approaches have made it increasingly difficult to distinguish the phenomenon of plasticity from that of robustness, for three major reasons. First, plastic responses are mediated in part by robust mechanisms that lend adaptive precision to a given phenotypic response, while robust phenotypes are often underlain by plastic mechanisms, such as the dynamic feedback responses involved in maintaining homeostasis (Bateson and Gluckman, 2011). For instance, the ability to specify discrete mouth-form morphs in the polyphenic nematode, *Pristionchus pacificus*, appears to be facilitated in part by the mutation buffering capacity of the heat-shock protein, Hsp90 (Sieriebriennikov et al., 2017). Second, both plasticity and robustness operate in a highly context-dependent manner. And lastly, both share, in large part because of their mechanistic similarities, the same evolutionary implications, in particular with respect to their ability to enable the accumulation and release of cryptic genetic variation (CGV). We begin by discussing the developmental basis of plasticity and robustness.

### Hormones and Growth-Related Pathways in Plasticity and Robustness

A common context in which the mechanisms of developmental plasticity and robustness have been evaluated and compared are the growth responses of different traits as a function of nutritional conditions during development. Organisms adjust their growth in response to nutrient availability, and do so in part by prioritizing the allocation of resources to some structures over others. For example, body size varies continuously in response to nutrition in most organisms (and particularly in insects; Nijhout and Callier, 2015), and many traits such as limbs or wings increase their size correspondingly, i.e., proportionately to body size. At the same time, some structures are shielded from nutritional variation, such as insect genitalia and the central nervous system (CNS), whose development is considered to be highly nutrition insensitive (Cheng et al., 2011; Tang et al., 2011). In contrast, exaggerated secondary sexual traits, such as weapons or ornaments, are often extremely sensitive to variation in nutrition. Thus, organisms can be thought of as mosaics of structures that exhibit more or less pronounced levels of plasticity or robustness in the face of the same, singular environmental factor: nutrition. In recent years, the mechanisms underlying trait-specific differential growth have begun to be elucidated, with substantial focus on candidate gene and pathway approaches.

The insulin/insulin-like signaling (IIS) and target of rapamycin (TOR) pathways have been studied in great detail in this context. The IIS pathway is a well-known pathway that increases insulin/insulin-like peptides in response to increasing levels of nutrition (Mirth and Riddiford, 2007). In contrast, the TOR pathway responds to amino acid levels. Both pathways interact through diverse components to coordinately regulate organismal growth and together, are often referred to as the IIS/TOR signaling pathway (Mirth and Shingleton, 2012). Three aspects of this composite pathway are especially noteworthy. First, this pathway and its interactions with other pathways are highly conserved and robust themselves (mutations commonly result in lethality; White, 2003; Wu and Brown, 2006). Second, IIS/TOR signaling has been found to simultaneously promote both plastic and robust phenotypic responses during growth. Third, whether plastic or robust development manifests is heavily dependent upon developmental context, and can vary heavily on a trait-by-trait basis. For instance, in the rhinoceros beetle, *Trypoxylus dichotomus*, the insulin receptor mediates differential nutrition sensitivity across organs, enabling extreme nutrition sensitivity in the growth of horns, while maintaining low nutrition sensitivity, and thereby suppressing growth, in genitalia (Emlen et al., 2012). Similarly, in *Drosophila*, low expression levels of *Foxo*, a downstream growth inhibitor in the IIS/TOR pathway that is normally expressed under low nutrition conditions, is thought to maintain nutrition insensitivity in genitalia by rendering them unresponsive to nutritional status, enabling them to develop at near constant sizes regardless of nutrition availability (Tang et al., 2011). Conversely, *Foxo* has been involved in mediating exaggerated, non-linear horn growth in the dung beetle, *Onthophagus taurus*. In this system, high *Foxo* expression levels in horn tissue are thought to contribute to the regulation of the threshold that sets apart small horned, low nutrition males from large horned, high nutrition males (Casasa and Moczek, 2018). Another mechanism maintaining nutritional robustness through insulin signaling can be seen in the CNS. In *Drosophila*, the growth of the CNS remains robust during starvation by constitutively expressing *Jelly belly (Jeb)* in glial cells surrounding neuroblasts. *Jeb* binds to its receptor (anaplastic lymphoma kinase, *Alk*), which directly activates downstream components of the IIS pathway (via PI3-kinase; Cheng et al., 2011). Taken together, IIS/TOR signaling appears capable of simultaneously regulating nutritional plasticity and robustness, either by employing different pathway components in different body parts of the same individual, or by using the same pathway components differently in different taxa. In the process, both extreme trait exaggeration and shielding from nutritional fluctuations are enabled as a function of spatial context (i.e., different body parts in different locations), and amplified by context-dependent pathway interactions.

The two major insect morphogenetic hormones, the steroid ecdysone and the sesquiterpenoid juvenile hormone (JH), have also been implicated in the regulation of nutrition-responsive growth, both through their interactions with IIS/TOR and independently (Layalle et al., 2008). These interactions have been the subject of detailed reviews elsewhere (Jindra et al., 2013; Koyama et al., 2013). Here we would like to focus in particular on the context dependent nature of hormone functions and the downstream responses they induce. For example, ecdysone, which regulates both molting between instars and the onset of metamorphosis, exhibits a peak during the last larval instar that induces growth arrest and the transition between larva and pupa (Nijhout et al., 2014). At the same time, lower ecdysone levels promote imaginal disk growth earlier during *Drosophila* development (mid-third larval instar) and do so via 4E-BP, a member of IIS/TOR signaling (Herboso et al., 2015). Thus, ecdysone signaling, too, is able to both promote and inhibit nutrition-responsive growth depending on developmental timing and expression levels. How spatial context interacts with developmental timing, and how these interactions in turn facilitate both plastic and robust responses, remains unclear.

Finally, nutrition-responsive growth can be regulated as a function of biological sex. For example, in the stag beetle, *Cyclommatus metallifer*, both the sexual dimorphism and nutrition sensitivity of male mandibles is regulated by the interaction between JH and the gene *doublesex* (*dsx*), which mediates sex-specific trait development in somatic tissues (Gotoh et al., 2014). In this system, female mandibles are normally small and nutritionally robust; however, the combination of *dsx* knockdown with JH treatment increased both mandible length and nutrition sensitivity. In contrast, the same treatment in males decreased length (via *dsx*), yet experimental JH application is insufficient to restore the full length of the wild type mandible. This suggests that JH sensitizes both males and females to nutrition, but the sex-specific context (i.e., the interaction with female or male-specific *dsx* splice variants) alters JH sensitivity to regulate a robust phenotype in females and a plastic phenotype in males (Gotoh et al., 2014).

### Patterns of Gene Expression and Gene Regulatory Networks

Although the mechanisms of plasticity and robustness are starting to be elucidated utilizing candidate gene/pathway approaches, the rise in next-generation sequencing tools has allowed for the assessment of genome-wide patterns of gene expression, and the identification of novel candidate genes and pathways underlying a particular plastic or robust response. Furthermore, similar approaches enable the assessment of whether and how patterns of gene expression may be regulated by epigenetic mechanisms. Collectively, these approaches are providing evidence that both plastic and robust trait formation are facilitated by gene expression across hundreds to thousands of loci, operating in a highly context-dependent manner, and that robust phenotypes may be mediated by extensive plasticity on the level of gene expression, and *vice versa*.

In the horn polyphenic beetle, *Onthophagus taurus*, a recent study by Ledón-Rettig et al. (2017) investigated the genome-wide regulation of sex-specific development and its regulation through the transcription factor *dsx*. By carrying out RNAseq on *dsx* knockdown individuals and pairing it with a genome-wide analysis of Dsx-binding sites across different tissues, the authors were able identify the repertoire of genes whose expression appears to be regulated by Dsx. This *dsx*-mediated gene repertoire was found to be highly sex- and tissue-specific, i.e., different tissues exhibited largely non-overlapping repertoires of *dsx*-responsive genes, as did the homologous tissues of males and females, with the only exception being head horn tissue, wherein *dsx* instead acted as a genetic switch, with the male isoform promoting expression of the same genes in males that the female isoform inhibited in females. Lastly, *dsx* was generally found to both promote but also extensively inhibit sex-biased gene expression, again depending on sex and focal tissue.

Follow-up preliminary experiments on the same taxon have begun to examine the nutritional responsiveness of *dsx* target repertoires by replicating the same RNAseq approach – originally executed on animals experiencing optimal nutritional conditions – at low nutrition (Ledón-Rettig et al., 2017). Under these conditions, morphological sex differences in two focal tissues – head and thoracic horns – largely disappear, *dsx*RNAi has minimal phenotypic consequences, and both control and *dsx*RNAi males now approximate the appearance of wild type females. In partial contrast, genitalic development - at least among males - appears unaffected by nutrition, and *dsx*RNAi has similar phenotypic consequences in all males, causing incomplete genitalia formation. However, once the transcriptional underpinnings of *dsx* function at low nutrition were examined, a much more complex picture emerged. Paralleling the reduced morphological consequences of *dsx*RNAi to horn development at low nutrition, the *dsx*-mediated gene repertoire of head and thoracic horns was correspondingly reduced at low nutrition. Yet in nutritionally insensitive male genitalia, a very different pattern emerged. While 215 genes were found to exhibit significant differential expression in the genitalia of control high compared to low nutrition male morphs, *dsx* was found to inhibit the otherwise nutrition-dependent expression of an enormous number of genes (*n* = 367) in low nutrition males only. These results suggest that *dsx* function is generally highly sensitive to nutritional context, and that in male genitalia in particular it is buffering against a genome-wide transcriptional response to low nutrition. In other words, in *O. taurus dsx* enables robustness in genitalia size by plastically inhibiting the nutrition-dependent expression of an enormous number of genes across the genome. Clearly, where plasticity ends and robustness begins is, at least in this case, in the eye of the beholder.

Gene regulatory networks underlying plastic and robust phenotypes have further increased our understanding of the relationship between robustness and plasticity in development. In particular, the wing polyphenism of ants in the genus *Pheidole* provides an excellent example of a highly conserved network underlying a plastic phenotype. Depending on environmental cues, *Pheidole morrisi* individuals will develop either as winged queens or as wingless soldiers and workers (Abouheif and Wray, 2002). The developmental regulation of this polyphenism is mediated by the highly conserved wing patterning network, with the wingless phenotype resulting from the inactivation of this network at variable, caste-specific (i.e., soldier or worker) points. Interestingly, the precise point where the network is interrupted has diverged further across different species (Abouheif and Wray, 2002), suggesting that the robustness of the winged or wingless response can be achieved through many routes during evolution, as long as the network’s outcome is consistent.

At the same time that the developmental genetic mechanisms and genome-wide expression patterns underlying plasticity and robustness are being discovered, the field of epigenetics, which focuses on the mechanisms by which changes in gene expression occur without changes to the underlying sequence of DNA, has also gained increased significance in recent years. Epigenetic markings such as methylation and acetylation are particularly interesting given that they are widespread (plants: Feng et al., 2010; Feng and Jacobsen, 2011; fungi: Martienssen and Colot, 2001; animals: Feng et al., 2010) and can be extremely responsive to environmental conditions, as well as provide a potential non-genetic mechanism through which environmentally induced phenotypes can be inherited across rounds of cell division and, in some cases, generations (Szyf, 2015; Hanson and Skinner, 2016; but see Charlesworth et al., 2017). In particular, the role of epigenetic mechanisms in developmental plasticity has been most thoroughly evaluated in social insects (Yan et al., 2014). For example, honeybee larvae destined to become queens have been shown to exhibit lower levels of methylation compared to workers, and knockdown of the *de novo* DNA methyltransferase, *dnmt3*, results in a bias toward queen development (Kucharski et al., 2008). Additionally, honeybee royal jelly contains a histone deacetylase inhibitor (Spannhoff et al., 2011), raising the possibility that epigenetic processes may contribute to caste determination. However, only a small fraction of the honey bee genome is actually methylated (three orders of magnitude lower than humans; Lyko et al., 2010) and several other insects lack either one or two DNA methyltransferases (e.g., Diptera lack DNMT1 and DNMT3, while Lepidoptera lack DNMT3; Bewick et al., 2017). Additionally, a recent study by Thomas et al. (2018) found several independent losses of epigenetic machinery across the arthropods and higher levels of DNA methylation in hemimetabolous compared to holometabolous insects, suggesting that the role of methylation in Hymenopteran development may not be representative of insects or arthropods at large. Thus, further investigation of holometabolous insects that show relatively high DNA methylation levels and corresponding plastic phenotypes is needed to better evaluate the nature and significance of epigenetic mechanisms in the regulation of plasticity.

In combination, the case studies presented here demonstrate that whether investigated at the level of hormones, genes, or genetic and epigenetic regulatory pathways, the nature of plasticity and robustness is characterized by (*i*) reciprocity, with plasticity being dependent on robust regulatory responses, and *vice versa*, as well as (*ii*) high context dependency across levels of biological organization. As a result, attempts to characterize mechanisms for the development of robustness separately from those for plasticity can be viewed as a largely unproductive endeavor.

### Evolutionary Consequences of Plasticity and Robustness for Developmental Systems

The evolutionary implications of developmental plasticity have been subject to intense debate (reviewed in West-Eberhard, 2003; Pfennig et al., 2010; Moczek et al., 2011). By comparison, the evolutionary implications of robustness in development have received less attention (but see e.g., Queitsch et al., 2002). However, the similarities in mechanistic underpinnings highlighted above, from signaling pathways to hormones to gene networks and transcriptional repertoires, suggest that it is increasingly difficult to separate the development of robustness from that of plasticity. Does the same also apply to their respective evolutionary implications?

To address this question, we would like to focus first on the phenomenon of cryptic genetic variation (CGV). Genetic variation is said to be cryptic when genotypic differences exist among individuals that have the potential to result in phenotypic differences through development, but fail to do so under some or most environmental conditions (Perry et al., 2010; Paaby and Rockman, 2014). Cryptic genetic variation can accumulate in populations when mechanisms exist that buffer the exposure of novel genetic variants. Further, CGV has the potential to become evolutionarily relevant when released, generally though the appearance of novel environmental conditions or stressors, thereby altering the nature and degree of phenotypic variation for selection to act upon, potentially altering the speed and direction of subsequent evolutionary changes (Paaby and Rockman, 2014).

Both plasticity and robustness are now understood to have the potential to contribute to CGV. When plastic responses to environmental conditions are underlain by modular, environment-specific gene expression, then only genetic variation present among individuals that experience the respective inducing environment will be phenotypically visible (Snell-Rood et al., 2010, 2011). Therefore, mutational variation contained in gene copies residing within individuals that do not experience the inducing environment pass unscreened by selection into the next generation. This phenomenon, known as relaxed selection, on one side hinders adaptation by slowing the speed with which beneficial mutations are able to spread to fixation within a population. On the other side, it may enhance adaptive evolution in situations when inducing environments become more frequent or constant, thereby exposing all previously cryptic genetic variation to the full strength of selection (Van Dyken and Wade, 2010; Ledón-Rettig et al., 2014). Results from diverse empirical and theoretical studies now corroborate the general validity of this framework (reviewed in Snell-Rood et al., 2010), yet the significance of plasticity-mediated CGV in evolutionary diversification largely awaits empirical assessment.

Mechanisms involved in maintaining robust and uniform phenotypic outcomes are similarly predicted to result in the accumulation of CGV. For example, heat shock proteins in both *Drosophila* and *Arabidopsis* act as chaperones that adjust incorrectly folded proteins into their proper conformation, thereby allowing small differences in coding regions to give rise to identical tertiary structures (Rutherford and Lindquist, 1998; Queitsch et al., 2002). As such, heat shock proteins conceal small but potentially heritable differences among genotypes without allowing them to manifest in functional differences under most sets of circumstances. However, when heat shock protein function is compromised, this cryptic variation becomes phenotypically expressed and selectable. Experimental studies on diverse organisms (including insects, plants and fungi) have documented a role of heat shock proteins as capacitors for CGV (Rutherford and Lindquist, 1998; Queitsch et al., 2002; Cowen and Lindquist, 2005) and suggest that this capacitance may play significant roles in the evolution of natural populations (e.g., Rohner et al., 2013). Taken together, both plasticity and robustness in development have the potential to influence levels of selectable variation in nature by biasing which genetic variants, and how frequently, become phenotypically expressed, and which do not. However, whether and how plasticity and robustness interact and possibly exacerbate, bias, or hinder the accumulation of cryptic genetic variation remains unexplored.

The second phenomenon we would like to examine briefly concerns the added levels of modularity contributed to by the regulatory mechanisms that underlie plastic and robust development. This argument has been made extensively in regards to the evolutionary implications of plasticity: plasticity adds developmental degrees of freedom by which organisms may adapt, populations may diverge, and species may form in the evolutionary process, thereby enhancing evolvability (Pfennig et al., 2010; Moczek et al., 2011). However, this argument can easily be extended in the light of our growing understanding of the extensive similarities between the developmental processes that underlie plasticity and those that enable robust developmental responses to environmental fluctuation. Allowing the same gene, pathway, or hormone to function as facilitators of plastic or robust development depending on context (e.g., body part, sex, social conditions, or season) enhances modularity in development and evolvability in diversification. For example, in the study by Ledón-Rettig et al. (2017) mentioned above, gene repertoires targeted by Dsx were found to greatly differ across tissues and sexes, a likely ancestral condition prior to the origin of the first horns. Following the origin of horns, Dsx rapidly evolved a horn-specific gene repertoire able to regulate plastic horn growth in response to nutrition in males, and highly robust horn inhibition in females (Ledón-Rettig et al., 2017). At the same time, *Onthophagus* species diversified dramatically in regards to both nutrition-dependent horn expression and degree and polarity of sexual dimorphism. Here, the large number of preexisting Dsx-binding sites across the genome likely facilitated the initiation, fine tuning, and diversification of novel interactions between Dsx and other pathways during the origin and diversification of horns.

More generally, this perspective emphasizes that plasticity and robustness do not just enable and complement each other in development, but may also synergize their respective contributions to the evolutionary process. This may best be expressed through the accumulation and release of cryptic genetic variation, or the evolution of modularity and its consequences for evolvability.

## Looking Outward: When Developmental Systems Shape or Determine Environments

In the previous section, we selected a variety of examples illustrating the means by which organisms experience, interpret, and respond to their environments. In each case, we showed how organisms can modulate, e.g., patterns of gene expression to allow their traits to best suit those environments, either by making developmentally plastic adjustments to morphology, physiology, and behavior, or by robustly maintaining those phenotypes, and suggested that these responses, or lack thereof, can be critically important factors in shaping evolutionary outcomes. In these examples, the arrow of causality pointed largely from the environment to the organism. However, in many cases, the arrow of causality can point from environment to the organism and back again. In this section, we discuss the nature and extent of this reciprocity. First, we discuss how early environmental inputs can induce plastic or robust environmental responses in organismal traits, and how these responses can alter the subsequent environmental conditions that organisms experience. Second, we explore how organisms, rather than adjusting their traits to the prevailing local environment, are capable of altering that environment directly through the process of niche construction. Finally, we discuss how host-associated microbiota comprise a highly complex but extraordinarily intimate feature of developmental environments, and may serve as an alternative route for the expression of plasticity or maintenance of robust developmental outcomes in their host.

### Environmental and Somatic Selection

During their development, many organisms experience challenging or sub-optimal environmental conditions. These organisms may respond to their local environment through coordinated plastic changes to morphological, physiological, and behavioral traits, aligning their phenotype more closely with the prevailing environment and maintaining or enhancing their fitness (see above for discussion). However, for many organisms, challenging environments need not be static features (Moczek, 2015). Rather, environments can be determined and selected through the mechanisms of plasticity, and this plasticity is often enabled by highly robust developmental processes. In this section, we explore the reciprocity between organism and environment by focusing on how organisms modulate their development and behavior in response to early environmental challenges, and discuss how these responses influence the subsequent environmental conditions in which organisms find themselves.

One direct means by which environmental selection can be achieved is through ecologically responsive habitat choice. For many highly motile organisms, this can be expressed through activational plasticity, an immediate response to environmental stimuli in which an organism shifts, e.g., its spatial location (*sensu* Snell-Rood, 2013), with daily or seasonal adjustments made in response to mates, predators, temperature, or food availability (Lidicker and Stenseth, 1992). Such responses are often underlain by highly robust neuroendocrine systems that reliably integrate environmental stimuli with coordinated behavioral phenotypes.

Alternatively, an organism’s response to the habitat in which it finds itself can be underlain by developmental behavioral plasticity (*sensu* Snell-Rood, 2013). The expression of such plasticity is a slower process than the expression of activational plasticity, requiring a period of environmental sampling but resulting in highly integrated phenotypic responses and coordinated changes in the subsequent development of organismal traits. For instance, through the trial-and-error process of learning, organisms express exploratory behaviors that enable them to broadly sample a range of environments. Through accumulated experience, behaviors or environmental states that are most beneficial to the organism can be somatically selected, refining and reinforcing neural networks during development in ways that may bias future environmental interactions (Luo and O’Leary, 2005; Snell-Rood, 2013). In some cases, plasticity in learning can lead to robustness in phenotype expression: for example, cabbage white butterfly (*Pieris rapae*) females with experience searching for host plants in novel or otherwise complex environments allocate more resources per offspring, generating offspring with larger bodies and wings that may enhance survival and fitness in these challenging environments (Snell-Rood et al., 2013). In addition to learning, developmental behavioral plasticity can be expressed when organisms undergo dramatic shifts between one or more discrete, alternative, developmentally plastic morphs. For instance, the presence of predators, low host plant quality, and high conspecific densities all induce female aphids to reliably and transgenerationally bias offspring to develop wings and disperse to a new host plant (Vellichirammal et al., 2017). Similarly, in response to increased population density and thus local competition for resources, *Schistocerca* locusts undergo phase transition from a cryptic, solitarious morph, to a highly gregarious, swarming, and dispersive morph (Pener and Simpson, 2009). As in the aphids, this dispersal polyphenism can be mediated transgenerationally through maternal epigenetic effects (Miller et al., 2008). Together, these examples illustrate how environmental features, however, transient, can induce plastic responses that range from short-term adjustments in behavior, to highly canalized, coordinated shifts in suites of morphological, physiological, and behavioral traits.

Historically, much thinking about plasticity has viewed developmental responses to the prevailing environment as being largely determinate (see Snell-Rood, 2012 for review), with environmental inputs eliciting changes in gene expression that correspond to particular phenotypes favored and refined by previous bouts of selection, such as in the dispersive and non-dispersive morphs discussed above. However, environmental and somatic selection is not limited to such strategies, but can additionally be expressed through the ways in which organisms phenotypically accommodate themselves to novel environments. Phenotypic accommodation refers to the ways in which developmental systems readily integrate environmental inputs through the coordinated and functional adjustment of their morphology, physiology, and behavior (West-Eberhard, 2005). These adjustments, when made in response to cues received in early development, can bias the future environments in which organisms find themselves for the remainder of their development and, in some cases, into adulthood. Prominent examples include the ways in which fish jaw bones and muscles (Wainwright et al., 1991; Mittelbach et al., 1992) and amphibian guts (Ledon-Rettig et al., 2008; Ledón-Rettig et al., 2010; Bloom et al., 2013) adjust their physiology and morphology in order to better utilize their particular dietary environments. In each case, these functional, non-random, and well-integrated phenotypes are enabled by highly canalized, conserved core processes that, through their exploratory behavior and later refinement through the process of somatic selection, are able to coordinately adjust developmental outputs in response to environmental context (see Gerhart and Kirschner, 2007). Importantly, these feedbacks between development and environment, and the adaptive fits they generate, may enable future phenotypic evolution. For instance, rearing ray-finned fishes in the genus *Polypterus* in a primarily terrestrial environment leads to the plastic induction of skeletal features and locomotive behaviors consistent with those of early terrestrial stem tetrapods, suggesting that phenotypic accommodation may have partially enabled the evolutionary transition of limbed vertebrates from water to land (Standen et al., 2014).

Collectively, these examples illustrate the highly constructive nature of organism-environment interactions during development, in particular how early environmental experiences shape patterns of phenotype formation that in turn select the subsequent environmental conditions that organisms experience. Throughout, we discussed how organisms are able to adjust aspects of their phenotype during environmental selection, primarily relying upon endogenous processes that are both highly exploratory and reinforced by somatic selection (Gerhart and Kirschner, 2007). Importantly, in each case, organisms attempt to better fit themselves to the functional demands imposed by their environment. However, a comparable fit between organism and their environment can also be achieved in fundamentally different ways, exemplified by niche construction, which we turn to next.

### Niche Construction

At the same time that organisms can plastically respond to environments through functional adjustments that better suit them to prevailing environmental conditions, organisms can also directly shape and bias their environments toward particular adaptive states through the phenomenon of niche construction. Niche construction refers to the modifications that organisms make to both abiotic and biotic selective environments, and is mediated by a wide array of physiological and behavioral phenotypes (as described in Odling-Smee et al., 2003). These modifications can range from the direct perturbation of environments, including the production of physical structures like nests or the chemical modification of soils and potential food sources, to relocation to novel habitats and social environments (Schwab and Moczek, 2017). Although habitat selection and learning (see above) have been considered forms of niche construction by previous authors (e.g., as relocational niche construction; Day et al., 2003; Odling-Smee et al., 2003), here we focus on the means by which individuals directly perturb, rebuild, and reorganize their environments, and the consequences of these modifications for our understanding of plasticity and robustness.

Conceptually, the relationship between developmental plasticity, robustness, and niche construction may best be conceived as being both reciprocal and interdependent. At one level, niche construction may be perceived as an alternative route from plasticity in how an adaptive fit between organism and environment is generated (Moczek, 2015). Whereas plasticity results from alterations to developmental, physiological, and life history traits that better suit those traits to the environment, acts of niche construction have the effect of altering the environment in ways that buffer the organism and its prevailing traits. Importantly, the nature of these interactions suggests that niche construction could be perceived as a robustness or canalizing mechanism not unlike the heat shock proteins introduced above, enabling the traits of organisms to follow their normal developmental trajectory and promoting normal growth and scaling relationships in environmentally sensitive traits. At another level, niche construction may be dependent upon the environmentally responsive nature of developmental systems that enable niche constructors to sense, evaluate, and adaptively adjust their environments (Schwab and Moczek, 2017).

That organisms engage in a variety of niche constructing behaviors is clear from a wealth of data on their natural history, and in some cases the potential physiological, developmental, and fitness contributions of niche construction are at least somewhat understood. For instance, the digging and tunneling behaviors of earthworms modify the surrounding soil in ways that increase its ability to capture and retain rain water, reduce its clay fraction, facilitate gas exchange, and increase its nutrient content due in part to the concentration of nitrogen and phosphorus found in worm excrement (as discussed in Turner, 2016). Similarly, it is well known that constructed dwellings, such as the nests of birds, the galls of wasps, and the tents of caterpillars, can promote developmental robustness by insulating individuals from environmental stressors such as adverse temperature conditions or predation (Joos et al., 1988; Abrahamson et al., 1989), findings that are consistent with expectations derived from ecological and population genetic models of niche construction (Laland et al., 1996, 1999). However, the nature of and degree to which environmental modifications affect phenotype formation during development is poorly understood, limited to only a few model taxa where niche constructed modifications can be experimentally manipulated without compromising the survival of the focal organism.

**FIGURE 1:**
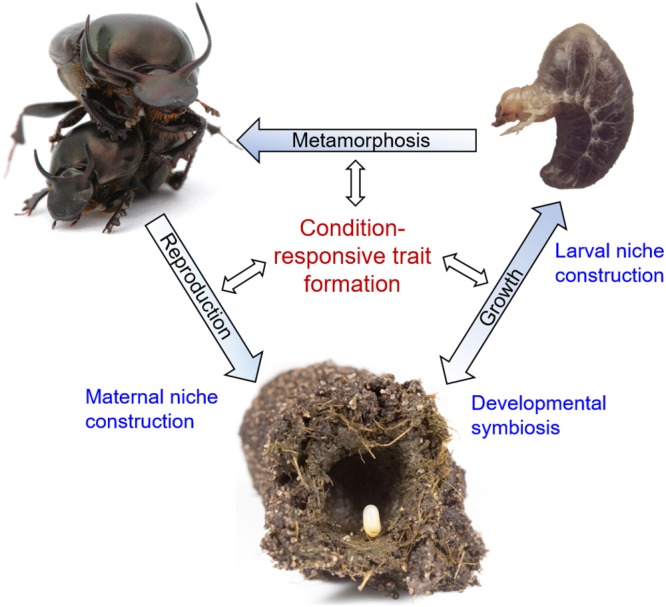
The reciprocally causal and constructive nature of developmental plasticity and robustness, placed within the life cycle of *Onthophagus* dung beetles. Blue font denotes processes that shape the environmental conditions experienced by individual beetles during their development, thereby affecting condition responsive trait formation at any stage of the life cycle (black font). Shown are, starting at the bottom, an *Onthophagus* egg positioned on a maternally derived fecal pellet – the pedestal – within a maternally constructed brood ball made of cow dung. Mutualistic relationships with gut endosymbionts (“developmental symbiosis”) shape the nutritional conditions experienced by growing larvae, which are further influenced by larvae’s own abilities to influence brood ball consumption through the process of “larval niche construction.” Larvae **(top right)** in turn adjust growth and late-larval trait proliferation leading into metamorphosis depending on the nutritional environment created during preceding stages of the life cycle. Lastly, adult beetles undergo maturation that once again depends on environmental circumstances, including the production of brood balls and pedestals (“maternal niche construction”) that influence the developmental environment experienced by their offspring. Images taken by Alex Wild **(top left)**, Sofia Casasa **(top right)**, and Guillaume Dury **(bottom)** and presented here with copyright holder’s permission.

One of the few studies involving the direct manipulation of niche construction has recently emerged through the study of dung beetle larvae. In *Onthophagus* dung beetles, mothers lay individual eggs within subterranean dung (‘brood’) balls, comprising all of the nutrition available to offspring during their development. As larvae, *Onthophagus* have been observed to engage in a wide array of putative niche constructing behaviors within the brood ball microenvironment (Figure 1, bottom), including feeding, defecating, and then re-feeding on dung, redistributing brood ball content, repairing the brood ball where maternal construction is inadequate, and forming a complex pupation chamber from dung fibers and the beetle’s own feces (Schwab et al., 2017). By experimentally inhibiting the extent to which *Onthophagus* larvae could directly modify the brood ball niche, Schwab et al. (2017) found that these niche constructed modifications were critical to both promoting normal growth outcomes and enhancing reproductive behaviors associated with fitness (i.e., brood ball size and number) in multiple beetle species. Intriguingly, inhibiting niche construction also altered scaling relationships in a number of traits, including eliminating or severely diminishing the expression of sexual dimorphism among male and female tibia in all species examined (Schwab et al., 2017). Although the presence and degree of sexual dimorphism is known to be environmentally contingent (as in Bonduriansky and Rowe, 2005; Bonduriansky, 2007; Allen and Miller, 2017), this finding extends earlier studies on the plasticity and condition-dependence of sexually dimorphic traits to the phenomenon of niche construction. Finally, Schwab et al. implicated the dung beetle external rumen, established when larvae spread their feces and thus maternally inherited gut microbiota throughout the brood ball chamber, as a possible mechanism of niche construction. In combination, this study suggests that niche construction may be a normal component of dung beetle development, lending robustness to both morphological traits and fitness. Evaluating the degree to which these findings from dung beetles are generalizable, as well as the extent to which niche constructing behaviors are themselves plastic with respect to prevailing environmental conditions, will be critical for better understanding both the nature of the organism-environment relationship and the interdependencies of plasticity and robustness.

Thus far, we have discussed the diverse ways in which individual organisms select, interact with, and even modify their own environments, as well as how these interactions result in, or are facilitated by, plasticity and robustness. Importantly, it is becoming increasingly clear that what comprises the individual in development, as well as in evolution, is very much a product of intimate partnerships between that individual and their microbiota (McFall-Ngai et al., 2013). In the next section, we explore how an organism’s expression of plasticity or robustness is often a function of the teams of microbial partners that live in, on, and around it.

### Host–Microbiota Interactions

Thus far, we have explored how the phenotypes of multicellular organisms are regulated by developmental mechanisms and processes that can simultaneously be characterized by both robustness and plasticity, and discussed how the expression of a robust or plastic phenotype is often itself the product of a highly reciprocal dialog between an organism and its external environment. Yet what is meant by the external environment, or what aspects of that environment are viewed as being critical in phenotype formation, has dramatically expanded in recent years with the advent of high-throughput sequencing technology. In particular, the newfound ability to taxonomically characterize the complex microbial communities found in and on the cells and organs of multicellular hosts, as well as to resolve these communities at a metagenomic and metatranscriptomic level, has led to a greater understanding of the ways in which environmental microbes can interact with and shape phenotypic variation in their hosts (McFall-Ngai et al., 2013). These findings, and the multiple levels of biological organization that microbiota appear to functionally influence (Gilbert et al., 2012), has led to speculation that hosts and their associated microbes may best be thought of as teams known as holobionts (Rosenberg et al., 2007), with selection acting on the composite genetic variation of host and symbionts that is contained within the hologenome (Zilber-Rosenberg and Rosenberg, 2008).

If true, this would suggest that developmental symbiosis, the concept that developing organisms are constructed and supported, in part, by interactions that occur between the host and its persistent communities of microbiota (Gilbert et al., 2015), may be the rule rather than the exception in development and evolution (Moczek, 2015). Indeed, such interactions begin at the earliest stages of embryogenesis; for instance, the obligate intracellular symbiont *Wolbachia* mediates cytoplasmic incompatibility across invertebrate taxa (Werren et al., 2008) and has been shown to influence the establishment of anterior-posterior polarity in nematodes (Landmann et al., 2014). Furthermore, symbionts have been shown to provide defense for developing embryos, generating compounds that inhibit the growth of pathogenic fungi in both invertebrates (e.g., in the shrimp, *Palaemon macrodactylus;* Gil-Turnes et al., 1989) and vertebrates (e.g., in the sea turtle, *Eretmochelys imbricata*; Sarmiento-Ramírez et al., 2014). During post-embryonic development, microbial symbionts have been shown to influence tissue and organ formation in diverse contexts, such as the light organ of the bobtail squid, *Euprymna scolopes* (see below; McFall-Ngai, 2014), as well as the guts of mice and zebrafish (Stappenbeck et al., 2002; Rawls et al., 2004), while also promoting settlement in planktonic marine invertebrate larvae (e.g., sponges: Whalan and Webster, 2014; tubeworms: Shikuma et al., 2014). Conversely, depriving organisms of their normally acquired developmental symbionts has been shown to compromise growth and development rate in diverse insect groups such as hempiterans, beetles, and flies (e.g., Storelli et al., 2011; Salem et al., 2013; Schwab et al., 2016), and has been linked to the etiology of certain disease states in humans (reviewed in Clemente et al., 2012). Therefore, developmental symbionts, through their co-evolutionary relationships with their hosts, can impart robustness upon and play an instructive role in normal development. Importantly, hosts are not only reliant upon interactions with symbionts to maintain robust developmental outcomes, but in some cases rely on these symbionts to mediate plastic responses to environmental stressors. For instance, the heat tolerance of plants ranging from cacti to maize is regulated in part by their fungal symbionts, which produce Hsp90 inhibitors capable of activating the plant heat shock response under severe temperature stress (reviewed in Gilbert et al., 2010).

As with other mechanisms of robustness or plasticity, benefits of developmental symbionts can be highly context-dependent. For instance, in *Onthophagus* dung beetles, mothers passage their gut microbiota to offspring by laying each egg on a pedestal made of her own feces (Estes et al., 2013). By experimentally manipulating the presence or absence of pedestal microbes, Schwab et al. (2016) have shown that the presence of these microbes not only increases growth while simultaneously decreasing development time (both positively correlated with fitness in insects; Kingsolver and Huey, 2008) under benign conditions, but that these benefits are disproportionately enhanced under ecologically relevant temperature and desiccation stressors.

Just as the phenomena of environmental selection and niche construction are characteristically constructive and reciprocal interactions between individuals and their environments, so too are the interactions between developing hosts and their symbionts. For instance, the morphogenesis of the bobtail squid light organ is induced through a series of reciprocal interactions with the luminous bacterium, *Vibrio fischeri*. This process begins when *V. fischeri* cells attach and aggregate along the mucociliary membranes of the light organ superficial epithelium, inducing cellular and transcriptomic changes in the host tissue (reviewed in McFall-Ngai, 2014). In particular, host epithelial tissues are induced to express a chitinase that hydrolyzes chitin polymers in the mucus into chitobiose, priming the symbiont and producing a chemoattractive gradient along which *V. fischeri* migrates into light organ tissues (Kremer et al., 2013). This reciprocity continues when *V. fischeri* cells populate the crypts of the light organ and induce their morphogenesis. Specifically, host receptors along these crypts sense cell wall components of *V. fischeri*, including lipopolysaccharide and peptidoglycan (Koropatnick et al., 2004), resulting in the apoptosis and regression of the ciliated epithelium that enabled colonization (McFall-Ngai, 2014). It is important to note that this reciprocity, in turn, is underlain by the same kinds of environmentally responsive processes that are characteristic of the plastic development described in earlier sections, and that here too, developmentally plastic responses are facilitated by robust responses and *vice versa*.

## Revisiting Plasticity and Robustness in Light of the Organism-Environment Relationship

Biologists have long been fascinated by the presumed oppositional nature of stasis and change in development and in evolution, and it may therefore not be surprising that the roles of plasticity and robustness in phenotype formation are so often viewed as in opposition, or at least as extremes along a continuum. This dichotomization is not limited to the concepts of plasticity and robustness, but pervades biological thinking, as for instance in the parceling out of the genotype from the phenotype, and the organism from the environment. In this review, we have sought to address the biological reality of this mode of thinking. In the first part, we looked inward at the nature of the genes, pathways, and hormones that enable context-dependent development, and posited that the same mechanism can promote both plasticity and robustness depending on context, and that across levels of biological organization plastic and robust developmental processes are highly interdependent and able to contribute to evolutionary outcomes in similar ways. In the second part, we looked outward toward the organism–environment relationship through the lens of three sets of phenomena, i.e., environmental and somatic selection, niche construction, and host–microbiota interactions. Throughout we emphasized similar themes, i.e., that these interactions are commonly highly reciprocal and constructive (see Figure 1 for summary of these phenomena in dung beetles). Taken together, we find that plasticity and robustness transcend all aspects of development and levels of biological organization.

With these general findings in mind, the field of plasticity and robustness research presents many opportunities for future investigation. Looking inward, it will be critical to continue to investigate the endogenous mechanisms regulating the expression of plasticity and robustness at the level of individual traits, and to do so in a comparative framework and across hierarchical levels of regulation (e.g., from discrete genes to gene networks). Doing so will further clarify the interrelatedness and independence of mechanisms that promote plasticity and robustness, and more generally lead to a better understanding of how the spectrum of phenotypic variation observed in populations is shaped, biased, and constrained by developmental processes. Furthermore, evaluating how these mechanisms influence the accumulation and release of CGV, and the extent to which such variation acts as a substrate for evolutionary change, continues to be a question of great interest for *evo devo* studies.

Looking outward, it is becoming increasingly clear that phenomena such as behavioral plasticity, niche construction, and host–microbiota interactions all have the potential to dramatically influence phenotype formation. Understanding how frequently and substantially processes such as these influence heritable phenotypic variation in natural populations, and their role in shaping the direction and speed of evolutionary change, is a topic of great importance to the field of plasticity and robustness research. At the same time, it will be important to now look inward, and to understand how phenomena such as host–microbiota interactions and niche construction influence patterns of gene expression in hosts or in individual niche constructors, respectively, as well as the extent to which these processes may themselves influence the accumulation of CGV. Addressing these questions will require a highly integrative approach, studying phenotype formation across multiple levels of biological organization, and a greater appreciation for the reciprocity in and constructive nature of development.

## Author Contributions

DBS, SC, and APM contributed to the writing of the manuscript.

## Conflict of Interest Statement

The authors declare that the research was conducted in the absence of any commercial or financial relationships that could be construed as a potential conflict of interest.
